# The work of the European Union Reference Laboratory for Food Additives (EURL) and its support for the authorisation process of feed additives in the European Union: a review

**DOI:** 10.1080/19440049.2015.1116127

**Published:** 2015-12-03

**Authors:** Christoph von Holst, Piotr Robouch, Stefano Bellorini, María José González de la Huebra, Zigmas Ezerskis

**Affiliations:** ^a^ European Commission, Joint Research Centre, Institute for Reference Materials and Measurements (IRMM), Geel, Belgium

**Keywords:** European Union Reference Laboratory for Feed Additives (EURL), authorisation of feed additives in the European Union, analytical methods for feed additives

## Abstract

This paper describes the operation of the European Union Reference Laboratory for Feed Additives (EURL) and its role in the authorisation procedure of feed additives in the European Union. Feed additives are authorised according to Regulation (EC) No. 1831/2003, which introduced a completely revised authorisation procedure and also established the EURL. The regulations authorising feed additives contain conditions of use such as legal limits of the feed additives, which require the availability of a suitable method of analysis for official control purposes under real world conditions. It is the task of the EURL to evaluate the suitability of analytical methods as proposed by the industry for this purpose. Moreover, the paper shows that one of the major challenges is the huge variety of the methodology applied in feed additive analysis, thus requiring expertise in quite different analytical areas. In order to cope with this challenge, the EURL is supported by a network of national reference laboratories (NRLs) and only the merged knowledge of all NRLs allows for a scientifically sound assessment of the analytical methods.

## Introduction

The production of high-quality feed containing suitable feed materials and feed additives is a key element in modern livestock farming. Feed additives play an important role in animal nutrition, addressing different aspects such as feed safety, reduction of environmental emissions and sustainability of food production. Prior to placing these products on the European Union (EU) market, industry needs to apply for authorisation. Feed additives are quite different in terms of chemical composition. More than 1000 feed additives are currently authorised within the EU and important examples are vitamins, trace elements, amino acids, enzymes and probiotics (European Commission 2015).

Due to their key role in animal nutrition, these products have been used already for long time in the EU and since the beginning of the 1970s these products have been authorised according to Council Directive 70/524/EEC. After a thorough review of the application of this council directive taking into account scientific progress in the field of feed additives and the requirements from the basic food law, i.e. Regulation (EC) No. 178/2002 (European Communities 2002), the European Parliament and the Council adopted Regulation (EC) 1831/2003 (European Union 2003), specifying new rules for the authorisation of feed additives. An overview of the development of the European feed law focusing on feed additives is available in the literature (Simon & Klaus 2008). One of the key components of the revised authorisation procedure is the separation of risk assessment to be conducted by the European Food Safety Authority (EFSA) and the risk management that falls under the responsibility of the European Commission (EC). Risk assessment in this specific context is the scientific evaluation a feed additive, whereas risk management refers to the final decision on granting or denial of a request for authorisation taking into account the opinion delivered by (EFSA). Furthermore, a quite significant change has been introduced regarding the use of growth-promoting agents with the phasing out by 2006 of the authorisation of antibiotics other than coccidiostats and histomonostats.

Regulation (EC) No. 1831/2003 classifies feed additives into five categories, namely technological, sensory, nutritional, zootechnical additives and coccidiostats or histomonostats. The categories are further divided into 25 functional groups and an overview of this classification is given in Annex II of the abovementioned regulation.

Another new aspect introduced by Regulation (EC) No. 1831/2003 is the creation of the Community Reference Laboratory (CRL, now the European Union Reference Laboratory – EURL) that plays a pivotal role in the authorisation procedure and which did not exist in the former legislation. Moreover, Annex II of this regulation specifies that this reference laboratory is the EC’s Joint Research Centre (JRC). The main tasks of the EURL are (1) the maintenance of a repository of feed additive samples that applicants need to send to the EURL when asking for authorisation; and (2) the evaluation of analytical methods linked to the sought authorisation of the feed additive. Since the authorisation of feed additives is most often connected to specific conditions of use, such as target concentrations of the active substance in feed or specification of the composition of the feed additive, the primary objective of the EURL’s evaluation is to establish whether these methods are suitable for enforcement of the conditions of use by member states’ authorities within the frame of official control. The EURL is supported in this task by a consortium of national reference laboratories (NRLs).

In November 2014 the EURL celebrated its 10th anniversary allowing us to evaluate the new system introduced in 2004. The purpose of this paper is to present the key activities of the EURL and its role in the feed additive authorisation procedure supported by practical examples.

## The authorisation procedure of feed additives

**Figure 1.**
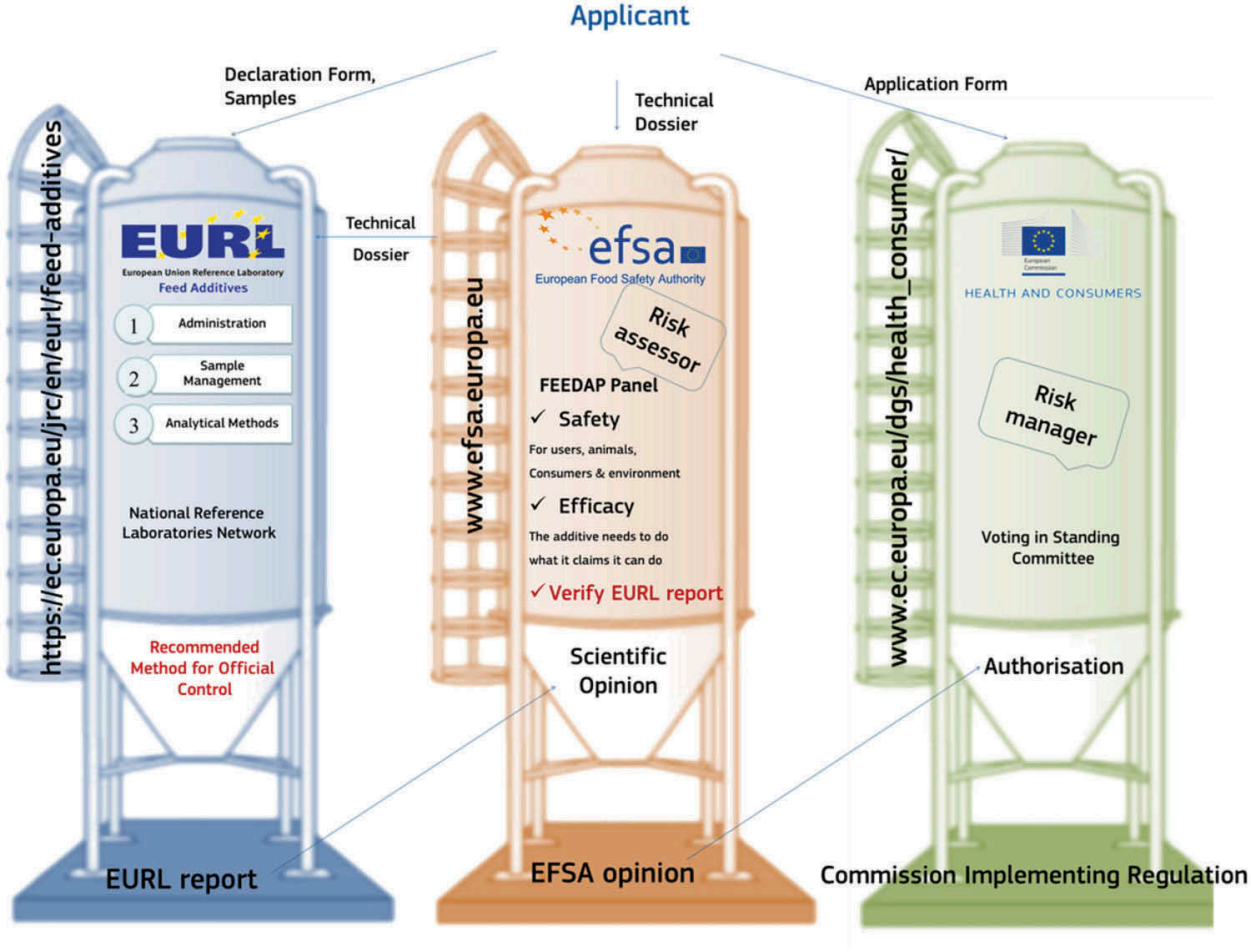
(colour online) Overview of the authorisation procedure for feed additives as outlined by Regulation (EC) No. 1831/2003, showing the interactions between the applicants and the European Union Reference Laboratory (EURL), the European Food Safety Authority (EFSA) and the European Commission. The main deliverables of the organisations involved are the EURL evaluation report of the analytical method, the scientific opinion of EFSA, and the regulation issued by the European Commission to grant or deny authorisation of the feed additive.

An overview of the whole authorisation procedure according to Regulation (EC) No. 1831/2003 is shown in Figure 1. This regulation requires that any feed operator that intends to place a feed additive on the EU market needs to follow a strictly regulated procedure. The administrative procedure consists of different phases which, depending on their respective roles, directly involve EFSA, the EC – specifically the Directorate-General Health and Food Safety (DG SANTÉ) – and the EURL. Whilst Regulation (EC) No. 1831/2003 established key elements of the authorisation procedure, additional regulations and various guidelines were issued by the EC to specify the rules applicable for presenting an application and the corresponding scientific dossier. Likewise, EFSA published corresponding guidance documents. Further details are given in Commission Regulation (EC) No. 429/2008 (European Union 2008), abbreviated here as the Application Regulation. This regulation specifies under chapter 2.6 of Annex II all the requirements that the applicant has to meet related to the methods of analysis of the feed additives. In addition, Commission Regulation (EC) No. 378/2005 (European Union 2005), as last amended by Regulation (EC) No. 885/2009 (European Union 2009a), defines the operation of the EURL, and is abbreviated here as the EURL Regulation.

Regulation (EC) No. 1831/2003 foresees various options for submitting authorisation applications, namely article 10(2) for existing products, article 4(1) for a feed additive or for a new use of a feed additive, article 13(3) for modification of authorisations, and article 14 for renewal of authorisations. The latter article reflects the aspect, that the authorisation of feed additives is limited to 10 years. The vast majority of several hundreds of applications submitted so far refers to the reauthorisation of existing feed additives under article 10(2) that have been previously authorised according to Council Directive 70/524/EEC. Whilst the deadline for submitting most of these applications was November 2010, the corresponding authorisation including evaluation of the dossier by EFSA and the analytical methods by the EURL is distributed over several years and is not finished yet. In the meantime these feed additives remain on the market, until a final decision concerning their authorisation according to Regulation (EC) No. 1831/2003 will be taken. In addition, an average of 30 applications per year, related to new products or new use of already authorised feed additives, is submitted under article 4(1). These applications are processed without delay.

## The sequence of the authorisation procedure

When submitting an application to the EC and the corresponding dossier to EFSA, the applicant needs to prove that feed additive samples have been sent to the EURL and a corresponding fee has been paid.

While Regulation (EC) No. 1831/2003 introduced the general requirement that the applicant sends three samples of the feed additive to the EURL and supports the cost of the operation of the EURL, details on the corresponding implementation of these provisions are given in the EURL Regulation. With respect to the fee, the EURL Regulation foresees a fixed amount that is comprised of two components, namely: one for the evaluation of the dossier and another for the maintenance of the sample repository. In addition, the EURL Regulation contains a number of case-specific exceptions to the payment requirement, reflecting the fact that for some of the applications the feed additive samples have already been provided and/or the analytical methods have already been evaluated via previous applications. For instance, for those applications under articles 4(1) and 13(3) that are linked to an already existing authorisation and if these new applications do not affect the analytical methods, the conclusions from the previous EURL evaluation also apply for the new application. Likewise, it is possible that the applicant has already sent feed additive samples to the EURL for previous applications. If both criteria are fulfilled, the applicant does not need to pay a fee and the EURL informs EFSA and the EC about the already existing EURL report, which is also valid for the new application. In addition, the EURL issues a letter confirming that the EURL has already obtained the feed additive samples.

In order to clarify the application-specific conditions with respect to the feed additive and the payment of the fee, the applicant has first to fill out the EURL declaration form (DF), as indicated in Figure 1. In this document the applicant provides key information concerning the application, such as the description of the feed additive, conditions of use including proposed legal limits, and details about the payment of the fee. Once these questions have been clarified, the EURL provides the applicant with confirmation letters with respect to the fee and the samples received. The applicant submits then an application to the EC via an application form (AF) available on the EC’s website (see http://ec.europa.eu/food/food/animalnutrition/feedadditives) and a dossier to EFSA. Once the application is considered valid by all parties, the EURL starts the scientific evaluation and within a defined time span of usually three months it issues a report concerning the suitability of the methods proposed for official control. Finally, and by considering the requests of the applicant and the scientific opinion issued by the authority, the EC presents a draft implementing regulation to the member states on authorising the product or on denying the application. If adopted by committee procedure, the authorisation is granted for 10 years or the application is denied. A correspondent official authorisation via a EC implementing regulation is issued, containing all the key details related to the product. As an example, the authorisation of the feed additive ‘Deccox’ with the active substance decoquinate and submitted under article 13 of Regulation (EC) No. 1831/2003 is shown in Table 1. The legal document contains all relevant information including the link to the corresponding EFSA opinion, legal limits of the active substance in feed and food, and the analytical methods recommended by the EURL for official control purposes. Once authorised, the feed additives are listed in the EU’s Register of Feed Additives (European Commission 2015), which is regularly updated by the EC.

**Table 1.** Example for the authorisation of the feed additive ‘Deccox’ containing the coccidiostat decoquinate as active substance.

Regulation	Commission Implementing Regulation (EU) No. 291/2014 of 21 March 2014 amending Regulation (EC) No. 1289/2004 as regards the withdrawal time and maximum residues limits (MRLs) of the feed additive decoquinate
Feed additive	Decoquinate (Deccox)
Active substance	Decoquinate
Reference to EFSA opinion	EFSA (2013)
Reference to EURL report in EFSA opinion	FAD 2013–009
Target levels of active substance in *feed* matrices	Feed additive: 60.6 g kg^–1^ Compound feed: 20 to 40 mg kg^–1^
Other components of the feed additive	Refined deodorised soya oil: 28.5 g kg^–1^, wheat middling: quantum satis 1 kg
Target levels of active substance in *food* matrices	1000 μg of decoquinate kg^–1^ of wet liver and wet skin + fat; 800 μg of decoquinate kg^–1^ of wet kidney; 500 μg of decoquinate kg^–1^ wet muscle
Short description of the method in *feed* matrices as evaluated by the EURL	Reversed-phase high-performance liquid chromatography with fluorescence detection (RP-HPLC-FL) – EN 16162
Short description of the method in *food* matrices as evaluated by the EURL	RP-HPLC coupled to a triple quadrupole mass spectrometer (RP-HPLC-MS/MS)

Note: Shown is the example for the authorisation of the feed additive ‘Deccox’ containing the coccidiostat decoquinate as active substance, submitted under article 13 (3) of Regulation (EC) No. 1831/2003. The feed additive was previously authorised by Regulation (EC) No. 1289/2004 and via the recent Regulation (EU) No. 291/2014 the conditions of the authorised product as proposed by the applicant are modified. Some key information provided in the regulation is given. The regulation also contains a link to the EURL website to have access to the EURL report.

The authorisation of the vast majority of feed additives is not linked to the applicant who submitted the dossier and requested the authorisation. This means that once the feed additive is authorised every feed operator can place the product on the market provided that this product complies with the criteria included in the corresponding regulation. In contrast, for zootechnical feed additives, coccidiostats and histomonostats and feed additives containing or produced from genetically modified organisms (GMOs) only the holder of authorisation indicated in the regulation can place the product on the market. Details for this aspect are given in articles 3 and 6 of Regulation (EC) No. 1831/2003.

## The interaction with the applicant

The described administrative procedure requires particular care to be taken by the applicant. In order to have a smooth and efficient process, the institutions involved developed several support tools. In this frame, the EURL maintains a specific website regarding its core statutory functions and activities. Furthermore, as foreseen by article 12 of the EURL Regulation, the EURL has issued a set of documents and forms published via a dedicated webpage (see https://ec.europa.eu/jrc/en/eurl/feed-additives) with the aim of guiding the applicant through the process and the interaction with the laboratory. In particular, the EURL has published a specific document named ‘Guidance for Applicants’ that describes the general procedure for the handling of the samples, the documentation and the fees for applicants seeking authorisation. The EC (see http://ec.europa.eu/dgs/health_food-safety/index_en.htm) and EFSA (see http://www.efsa.europa.eu/en/applicationshelpdesk/feedadditives.htm) have issued similar guidance on their respective websites. The EURL has also included in the website several technical recommendations for the correct implementation of the verification concept of analytical methods. The verification concept is required for all single-laboratory validated methods and will be explained below. Furthermore, and following the Treaty of Lisbon, a transparent and open EU public administration is considered as a fundamental right and an institutional principle. In this context, all the evaluation reports issued are published and freely downloadable. In addition, as specified in article 18.3.e of Regulation (EC) No. 1831/2003, even the methods of analysis will not be considered confidential. For this reason, any control laboratory – including a second feed business operator – can have access to them once the feed additives are authorised.

## The tasks of the EURL

The main duties and tasks of the EURL in the frame of feed additives authorisation are the following: (1) reception, storage and maintenance of the reference samples of feed additives sent by applicants; (2) scientific evaluation of the analytical methods submitted by applicants as regards their suitability for official control; and (3) drafting and submitting the evaluation reports to EFSA and the EC.

## Evaluation of analytical methods provided by the applicants

### General

All information that the EURL needs for carrying out its assessment is available in the AF and the dossier. Particularly, it includes the physical–chemical characterisation of the feed additive, its proposed conditions of use and details on the analytical methods. The latter ones relate mainly to the determination of active substances of concern in the relevant matrices, such as the feed additive, pre-mixtures, compound feedingstuffs, water and/or silage. For some feed additives, such as coccidiostats or carotenoids, MRLs in tissues are set, requiring additional methods of analysis for the determination of residues of these target analytes or metabolites in food products (i.e. eggs, milk, meat etc.). An example of this case is given in the evaluation report for the feed additive Monimax with the active substances monensin sodium and nicarbazin (EURL Feed Additives 2014). In other cases the feed additives consist of a complex mixture of different substances such as the natural product cassia gum (EURL Feed Additives 2012), which may be used as feed binder. Whilst there are no methods that allow the determination of the content of such a complex mixture in feed, quality criteria for the composition of the additive as such may be established by the corresponding regulation, for which the applicant submits suitable methods of analysis.

As the analytical methods are evaluated by the EURL against their suitability for official control purposes, the Application Regulation specifies that:

These methods shall meet the same requirements as those for methods of analysis used for official control purpose laid down in Article 11 of Regulation (EC) No 882/2004. This Regulation is on official controls performed to ensure the confirmation of compliance with feed and food law, animal health and animal welfare rules.

Moreover the Application Regulation requires that the applicants provide for each active substance/matrix combination (1) a standard operating procedure to describe the analytical method used and (2) a single-laboratory validation report to present the relevant method performance characteristics and to demonstrate that the method is fit for the intended purpose. In addition, (3) a verification report – showing that these performance characteristics are confirmed by a second independent expert laboratory – to demonstrate the transferability of this method to official control laboratories. When proposing a standard method, the applicant does not need to submit a single-laboratory validation report. However, the EURL may request experimental data showing that the proposed standard method is applicable to the analysis of the specific feed additive.

The applicants need to describe their ‘in-house’ developed standard operating procedure using the ISO 78-2 format and they should implement the recommendations of the ‘IUPAC harmonised guidelines for the single-laboratory validation of the methods of analysis’ (Thompson et al. 2002). The full list of performance characteristics to be investigated is prescribed in annex III of Regulation (EC) No. 882/2004 (European Union 2004). For the verification study, a reduced experimental design applies. In a few cases several applicants combined their authorisation requests and submitted a verification study consisting of a collaborative trial, where more than three laboratories provided results applying the same prescribed analytical method. Reproducibility was then the additional performance characteristic reported.

During the evaluation procedure the EURL may also recommend other methods than those included in the dossier. In particular, analytical methods that are available as national or international standards play an important role in the evaluation process. This aspect is linked to the method cascade of Regulation (EC) No. 882/2004 required for methods used within the frame of official control, and will be discussed below.

Upon reception of a new feed additive dossier, the EURL nominates a dossier rapporteur laboratory, experienced in the analytical field of concern, which will evaluate the information provided and will draft the evaluation report to be finalised within three months. An NRL as well as the EURL can act as a dossier rapporteur. The analytical methods are assessed against the ‘conditions of use’ of the feed additive such as target levels as specified in the register entry of the product proposed to the EC. The available experimental data are systematically reviewed and the performance characteristics, such as precision, recovery rate and LOQ, are thoroughly evaluated. When the available information is deemed insufficient or when unsatisfactory results are reported, the dossier rapporteur requests via the EURL additional information and clarification from the applicant. This may delay the finalisation of the report by one month or, when more time is needed to provide the supplementary information/clarification, even trigger the request of a clock-stop by EFSA. Once the initial evaluation report is ready it is reviewed and commented on by the network of NRLs. All comments are taken into account when drafting the final evaluation report, which is finally sent to the EFSA, to the EC and to the applicant.

### The verification concept

One of the major challenges of the EURL is the evaluation of the analytical methods proposed by the applicants against their suitability for official control given the fact that the majority of the methods are single-laboratory validation methods. While the presentation of a method with acceptable analytical performance characteristics in the applicant’s dossier may allow the EURL to conclude that the method in principle is fit for the intended purpose, it is very difficult to judge the question whether the method protocol is drafted in such a way that a national control laboratory can implement and apply the method without major problems. This is a well-known problem and method validation via an inter-laboratory study is therefore often considered as a gold standard. However, most of the applicants’ analytical methods have not been validated against this standard. In order to address this question, the EURL Regulation foresees under article 5(2) the option that in specific cases the EURL concludes in its evaluation report that either the method needs to be tested in another laboratory or even an inter-laboratory study needs to be organised. This work – leading to additional costs for the applicant and certainly to a delay of the whole evaluation procedure – may be justified in special cases, for instance when dealing with substances of an increased risk, but is not considered practical for the majority of the submitted applications. This topic was intensively discussed in earlier workshops with the network of NRLs in 2004, and based on these discussions the alternative concept of the verification study was put forward and was finally included as requirement under paragraphs 2.6.1.3 and 2.6.2.3 of annex II of the Application Regulation. The concept is based on the principle that in the case of a single-laboratory method, the applicant needs to transfer the method protocol to a second laboratory not involved in the development of the method. This second laboratory conducts the analyses on samples provided by the applicant according to the applicant’s method protocol and by applying an experimental design proposed by the EURL and available on the EURL website. This document also contains a form on which to report relevant method performance indicators obtained in the applicant’s laboratory and in the second laboratory. A key advantage of this approach is that these results from experiments demonstrating the transferability of the method should be already available when presenting the dossier, thus allowing the EURL to carry out the evaluation without asking further experimental work. By comparing the method performance profile in both laboratories, the EURL finally concludes whether the method protocol could be successfully verified in the second laboratory. The second laboratory verification became compulsory in 2008 and experiences until the present do not reveal major problems for the applicants when implementing this concept.

### The method cascade

Regulation (EC) No. 882/2004 (European Union 2004) lays down general rules for the performance of official controls to ensure the compliance with feed and food law, animal health and animal welfare rules. In concrete, article 11 of this regulation establishes a classification of analytical methods based on its standardisation level. According to that, a hierarchy of analytical methods, widely known as a ‘method cascade approach’, appeared for the first time in a legislative document, making it binding in all member states. Moreover, by including this concept of Regulation (EC) No. 882/2004 in the Application Regulation, the method cascade approach also applies to applicants when proposing a suitable method of analysis in their dossier.

In practice, the method cascade approach specifies that the highest status is given to those methods complying with EC rules, so called ‘Community Methods’ (European Commission 2009) and, whenever existing, they shall be used in the frame of official controls.

In the case that such methods do not exist, the methods falling in the following level in the cascade should be chosen. To this level belong those methods complying with internationally recognised rules or protocols or those agreed in national legislation. The regulation specifically mentions the European Committee for Standardisation (CEN), however also other standardisation bodies at international level fall within this group such as the International Organization for Standardization (ISO) or AOAC International. Furthermore, also methods from standardisation bodies at a national level or analytical methods established by national legislation are on this level.

Often the applicants propose for the feed additive purity criteria or a list of target identification criteria according to the European Pharmacopoeia (Council of Europe. European Directorate for the Quality of Medicines & Health Care 2015), Food Chemical Codex (The United States Pharmacopoeial Convention 2015) and/or Joint FAO/WHO Expert Committee on Food Additives (FAO 2015). The corresponding methods of analyses available in these publications are considered as standards and are therefore recommended by the EURL accordingly.

If none of the abovementioned methods is available, the third level to be chosen in the cascade approach refers to methods fit for the intended purpose or developed with scientific protocols and validated in a ring test in accordance with an internationally recognised protocol on collaborative trials such as ISO 5725 or International Union of Pure and Applied Chemistry (IUPAC) (Horwitz 1995).

If still no suitable methods from the higher levels are found, the last option given in the cascade approach allows the use of analytical methods that have been previously single-laboratory validated according to international harmonised guidelines such as the IUPAC Harmonised Protocol (Thompson et al. 2002).

In the light of the enforcement of this legislation, the use of standardised methods of analysis is of utmost importance to ensure the control in a harmonised way of the compliance with European legislation and thus to enforce regulatory requirements. However, the existing EC methods (European Commission 2009) currently used to carry out the control of feed additives in feedingstuffs are in many cases based on obsolete methodologies. Moreover, most of these methods just allow the measurement of a single analyte, while the trend nowadays goes towards multi-analyte methods and/or standards that still leave some freedom to laboratories of choosing between different types of instrumentation. In order to close the gap between the characterisation of the existing analytical methods for feed and the demands from member states’ official laboratories with respect to modern analytical methods, the EC has issued different mandates addressed to CEN. The objective of these mandates is to provide European standards for a number of banned antibiotics previously used as feed additives and for some of the currently authorised feed additives such as coccidiostats, organic acids, carotenoids and vitamins. The projects are carried out within the technical committee CEN/TC 327 (Animal Feeding Stuffs – Methods of Sampling and Analysis) and its workgroup 3 (Feed Additives and Drugs). More information about the structure, work programme and published standards of this technical committee are available on CEN’s website (see www.cen.eu/work/areas/food).

In line with this approach, the EURL performs additional tasks to foster the use and availability of analytical methodologies validated according to international guidelines. In essence, the EURL is reaching this goal in two ways. One way is through the recommendation of standard methods for official control when evaluating dossiers. This may include the request to the applicant to apply the standard to check whether this method works well for the specific feed additive under assessment.

Additionally, the EURL actively contributes to the standardisation process of analytical methods by acting as project leader for some of the items included in corresponding EC mandates given to CEN. For instance, in the last completed Commission Mandate M 382/2006, the EURL acted as project leader for the standardisation of a method for the analysis for semduramicin, which is one of the authorised coccidiostats. This method, which is based on LC, has been previously developed and single-laboratory validated by the EURL and it allowed the use of two different detectors, namely post-column derivatisation (LC-PCD-UV/VIS) (Gonzalez de la Huebra et al. 2011) and LC-MS (Gonzalez de la Huebra et al. 2012). Within the time frame of this mandate the EURL organised an international collaborative study involving expert laboratories in the field of feed analysis, followed by preparation of the final standard published under the identification number EN 16158:2012 (CEN 2012).

The EURL has also supported the organisation of an international collaborative study for the determination of phytase activity including statistical assessment of the results reported by the participating laboratories. This work led to the international standard published under the identification number EN ISO 30024:2009: (CEN 2009) and is the only standard in the field of the analysis of enzyme activity that can be applied to various phytase feed additives produced by different companies.

### Role of the network of national reference laboratories (NRLs)

The evaluation of the analytical methods is a quite challenging task, given the variety of the methodologies involved. In fact, quite different analytical techniques are applied in the field of feed additive analysis such as GC and LC, trace element analysis, microbiological counting method and enzyme activity analysis. From this list it is obvious that a single laboratory alone cannot conduct a scientifically sound evaluation for all types of analytical methods. In order to cope with this challenge, the EURL is supported by a consortium of NRLs contributing their specific knowledge in analytical science to the evaluation of the analytical methods. Moreover, NRLs play a quite important role in the evaluation exercise because they may be appointed as a rapporteur laboratory by the EURL responsible for the evaluation of the dossier and drafting the initial report. Furthermore, NRLs are requested that they comment on the initial report of each evaluation within the peer-review period foreseen in the EURL Regulation. The comments play a key role in the evaluation of the methods and it has happened several times that the EURL asked supplementary information from the applicant based on a specific comment received.

The NRLs are proposed by the different member states and appointed by the EC if the requirements are fulfilled. The list of the NRLs is given in annex II of the EURL Regulation.

## The feed sample repository

The samples collected by the EURL are appropriately stored in a sample bank according to the temperature conditions specified by the applicant. The samples are one of the key components along with the dossier and other documents that the applicant has to submit when applying for the authorisation of a feed additive. The samples may be used during the period of application by conducting an analysis to gain additional information for a comparison with the details presented in the dossier. In addition, the samples are available to national authorities for official control analysis. In this context, the EURL may further request additional reference samples/standards and feed/food test materials from the applicant. For some feed additives such as coccidiostats, the applicants need also to send the active substance in addition to the feed additive, which is in the case of coccidiostats a specific formulation, to the EURL. The reason is that to conduct the analysis the active substance is required for calibration purposes and not all these active substances are commercially available. When receiving a feed additive sample, the EURL conducts a thorough check of all the documents provided including the certificate of analysis, storage conditions and expiry date. Finally, a subsample of each feed additive is subjected to chemical analysis applying near-infrared, mid-infrared and Raman spectroscopy as a rapid non-destructive identification tool. Moreover, the EURL has used feed samples in the past for both purposes, namely (1) supplementary analysis of the composition of the product and the determination of traces from the production process and (2) the preparation of test material for the organisation of inter-laboratory studies.

## The achievements

This section summarises the main achievements of the EURL in the period 2004–14.

### Feed additive samples

A total of 700 reference feed additives samples are stored at the facilities of the EURL-FA. The samples are properly documented, kept at proper conditions, which are recommended by the applicants, and occasionally replaced (or shelf-lives extended) in the case of the expiration of the old samples.

### Categories of feed additives and analytical methods

Table 2 shows the number of EURL reports delivered in the period 2005–14, confirming that analytical methods related to dossiers from almost all categories/functional groups had to be evaluated. This demonstrates the huge variety of analytical methods given the fact that among these groups there are quite different feed additives such as microorganisms, enzymes, vitamins, amino acids, nutritional elements (selenium, cobalt, zinc, copper, nickel, manganese etc.) and various organic, inorganic and polymeric compounds.

**Table 2.** EURL evaluation reports on analytical methods in the period 2005–14: number of feed additives covered by these reports and distribution across categories and functional groups.

	Category		Functional group	Total
1	Technological	A	Preservatives	14
		B	Antioxidants	9
		C	Emulsifiers	13
		D	Stabilisers	11
		E	Thickeners	11
		F	Gelling agents	10
		G	Binders	7
		H	Substances for control of radionuclide contamination	1
		I	Anti-caking agents	6
		J	Acidity regulators	6
		K	Silage additives	19
		L	Denaturants	–
		M	Reduction of the contamination of feed by mycotoxins	8
2	Sensory	A	Colorants	22
		B	Flavouring compounds	43
3	Nutritional	A	Vitamins, pro-vitamins	28
		B	Compounds of trace elements	27
		C	Amino acids	25
		D	Urea and its derivatives	1
4	Zootechnical	A	Digestability enhancers	65
		B	Gut flora stabilisers: microorganisms	57
		C	Substances which favourably affect the environment	14
		D	Other zootechnical additives	37
5	Coccidiostats and histomonostats			30
			Total	464

### EURL reports

**Figure 2.**
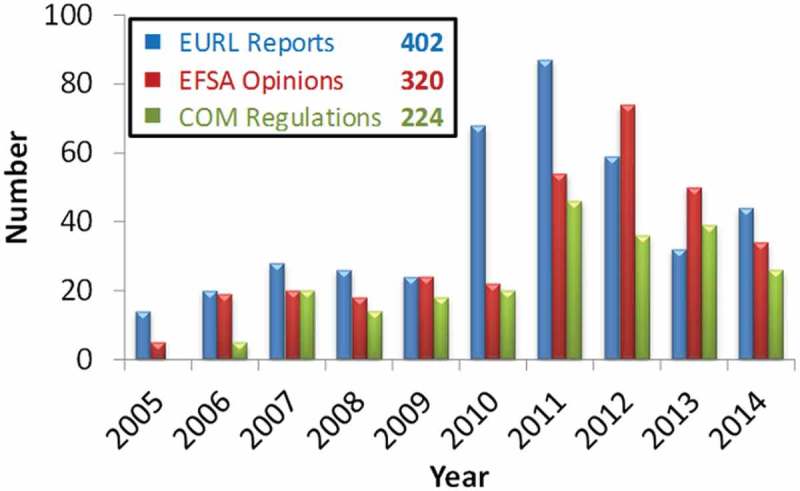
(colour online) Evaluation reports issued by the EURL-FA and their impact expressed in terms of number of EFSA opinions and European Commission regulations delivered per year and total number across all years.

In the period from 2005 to 2014, the EURL-FA evaluated around 490 dossiers and drafted 400 evaluation reports with the support of the NRLs. Figure 2 gives an overview of the number and trend of the reports issued. A significant increase in the number of the dossiers and reports was recorded in 2010, reaching a maximum of 124 dossiers evaluated and 87 reports issued in 2011. This increase marked the start of the reauthorisation exercise of the feed additives according to the new requirements imposed by Regulation (EC) No. 1831/2003. The difference between the number of dossiers and the number of the reports, which started to occur from 2010 onwards, is related to the fact that the EURL occasionally used to draft one evaluation report for several applications, similar in terms of analytical methodology recommended for the official control of feed additives. This mode of reporting is foreseen in article 3(5) of the EURL Regulation.

### EURL’s impact on EU policy

In the period from 2005 to 2014, EFSA issued more than 300 opinions where the executive summaries of the EURL evaluation reports were included. In the same period, more than 200 Commission Regulations for the authorisation of feed additives with the annexes containing recommended analytical methodology for official control were published. The trend of opinions and regulations issued from 2005, where the contribution of the EURL is reflected, is shown in Figure 2.

### Workshops and annual reports

The EURL organised in the period 2005–14 fourteen meetings (workshops) involving the consortium of NRLs and representatives from EFSA and DG SANTÉ. The events included an overview of the EURL annual activities, prospects for future work, as well as scientific, technical presentations and discussions. The topics discussed regularly are described in the workshop proceedings. In the period from 2005 to 2014, the EURL drafted nine annual reports reflecting the yearly achievements and work flow. The full reports are available from the EURL-FA webpage.

## Case study: analytical methods related to feed additives reducing mycotoxin exposure

Regulation (EC) No. 1831/2003 also foresees the option of establishing new categories or functional groups in order to respond to new requirements, also reflecting scientific progress. In 2009, the EC (European Union 2009b) introduced a new functional group in the category of technological additives, which were ‘substances for reduction of the contamination of feed by mycotoxins: substances that can suppress or reduce the absorption, promote the excretion of mycotoxins or modify their mode of action’. As pointed out in the recital of this regulation, the purpose for the use of these additives is to gain a favourable effect of feed containing specific mycotoxins such as aflatoxin B1 (AFB_1_) at concentrations even below the legal limit in feed as established by European legislation (European Commission 2003). This means that feed additives of this new functional group must *not* be used in order to feed animals with feed containing AFB_1_ *above* the legal limit. One important prerequisite for considering the approval of such a new functional group was the availability of results from experiments confirming that the determination of AFB_1_ in feed within the frame of official control needs to be unaffected by the presence of these new group of feed additives. In order to establish whether such an adverse effect exists, the EURL for mycotoxins conducted an extensive experimental study (Kolosova & Stroka 2012). The results of this investigation revealed that no significant differences of the analysed mycotoxin concentrations could be observed between feed with or without the added mycotoxin binder.

In between, various products have been authorised under this functional group, namely a specific microorganism strain capable of biotransformation of trichothecenes (European Commission 2013a), a fumonisin esterase capable of the biotransformation of fumonisins (European Commission 2014), and bentonite capable of binding AFB_1_ (European Commission 2013b).

The annexes of the authorisation regulations contain key information regarding the target content of the additives in feed, criteria for the characterisation of the product and corresponding analytical methods to be used for official control. An overview of these details is given in Table 3. Comparison of the information for the three different feed additives reveals that both the conditions of use and the methods of analysis are quite different:

**Table 3.** Authorisation of feed additives reducing the mycotoxin content in feed.

Feed additive	Regulation authorising the feed additive	Criteria included in the Regulation specifying the conditions of use	Method of analysis included in the Regulation to enforce the conditions of use of the authorisation
Microorganism strain DSM 11798 of the Coriobacteriaceae family	European Commission implementing Regulation (EU) No. 1016/2013	Minimum content: 1.7 × 10^8^ CFU kg^–1^ of complete feedingstuff with a moisture content of 12% and specific strain	*Enumeration* of microorganism strain DSM 11798 of the Coriobacteriaceae: pour plate method using agar supplemented with oxyrase. *Identification* of microorganism strain DSM 11798 of the Coriobacteriaceae: pulsed-field gel electrophoresis (PFGE)
Bentonite	European Commission implementing Regulation (EU) No. 1060/2013	Maximum content: 20 000 mg kg^–1^ feedingstuff with a moisture content of 12% Characterisation of active substance bentonite: ≥ 70% smectite (dioctahedral montmorillonite) < 10% opal and feldspar < 4% quartz and calcite AFB_1_-binding capacity (BC AFB_1_) > 90%	For the determination of bentonite in feed additive: X-ray diffraction (XRD) For the determination of BC AFB_1_ of the additive: adsorption test carried out in a buffer solution at pH 5.0 with a concentration of 4 mg l^–1^ for AFB_1_ and 0.02% (w/v) for the feed additive
Fumonisin esterase EC 3.1.1.87	European Commission implementing Regulation (EU) No. 1115/2014	Preparation of fumonisin esterase produced by *Komagataella pastoris* DSM 26643 containing a minimum of 3000 U g^–1^ Minimum content: 15 U kg^–1^ of complete feedingstuff with a moisture content of 12%	For the determination of fumonisin esterase activity: HPLC-MS/MS method based on the quantification of the tricarballylic acid released from the action of the enzyme on fumonisin B1 at pH 8.0 and 30°C

Conditions of use and analytical methods are as specified in the corresponding European Union regulations authorising these feed additives. CFU, colony-forming unit; U, 1 U is the enzymatic activity that releases 1 μmol of tricarballylic acid per minute from 100 μΜ fumonisin B1 in 20 mM Tris-Cl buffer pH 8.0 with 0.1 mg ml^–1^ bovine serum albumin at 30°C. The purpose of the methods of analyses is to enforce the conditions of use. However, to enforce the maximum content of bentonite, no method of analysis suitable for official control could be identified.

### Microorganism (feed additives identification number: 1m01)

For this additive, a minimum content in feed is specified which is expressed in terms of CFUs kg^–1^ feed. Furthermore, only a specific strain is authorised. For both criteria, corresponding methods of analyses: pulsed-field gel electrophoresis for the identification of the strain and an enumeration method for the quantification of CFUs in feed, have been identified and included in the regulation.

### Fumonisin esterase (feed additives identification number: 1m03)

For this additive, the minimum content is expressed as enzyme activity which is defined as the release of a specific amount of tricarballylic acid formed by the reaction between fumonisin and the enzyme at specific conditions. For the determination of enzyme activity, the quantification of the product is performed by applying liquid chromatography coupled to triple quadrupole mass spectrometry (LC-MS/MS).

### Bentonite (feed additives identification number: 1m558)

With respect to this additive, the corresponding regulation established a maximum content of 20 000 mg kg^–1^ in feed and specific characteristics of the feed additive. The latter aspect includes maximum and minimum values for the content of specific mineralogical parameters. In addition, a minimum value for the binding capacity of AFB_1_ was established at 90%, and this value can be measured by applying a specific experimental protocol. In this context, two aspects need to be emphasised. Firstly, the purpose of this method protocol is not to demonstrate efficacy of the feed additive but instead to establish whether a feed additive placed on the market under this specific authorisation regulation complies with the criterion of the binding capacity of AFB_1_ specified there. This is a key aspect for official control given the fact that (1) the authorisations related to this category are *not* holder-specific and (2) bentonite is a widespread used feed additive also for other purposes such as an anti-caking agent. Secondly, the binding capacity consists of an adsorption experiment conducted in an aqueous buffer solution at specific conditions that need to be scrupulously applied in order to obtain a result of analysis which is comparable with the target binding capacity. Therefore, the precise conditions in terms of the pH of the buffer solution and the concentration of AFB_1_ and the feed additive in the solution are included in the regulation authorising the feed additive.

## Conclusions

Feed additives are important components in modern livestock farming. However, prior to placing them on the EU market, they need to be authorised according to a procedure established by Regulation (EC) No. 1831/2003. In general, feed additives are authorised along with specific conditions of use such as legal limits. The enforcement of the conditions of use within the frame of official control requires suitable methods of analysis which are proposed by the industry when asking for authorisation. It is the main task of the EURL supported by a consortium of NRLs to carry out the evaluation and to establish whether the methods are fit for the intended purpose. The work presented in this paper showed not only the complexity of the task of the EURL but also a very typical example of the projects of JRC, reflecting its mission to provide EU policies with independent, evidence-based scientific and technical support throughout the whole policy cycle. Furthermore, it demonstrated that the successful method evaluation related to the applications was only possible with the support of NRLs. This strong network between the EURL and the NRLs allows a unique merging of expertise in analytical methodology at the European level. Whilst the evaluation of applications will remain the major task in future, further improvement of the EURL website is scheduled to facilitate the availability of protocols of analytical methods related to authorised feed additives.

## Disclosure statement

No potential conflict of interest was reported by the authors.
